# The Impact of Irritable Bowel Syndrome on Spine Surgery Outcomes: A Comprehensive Narrative Review

**DOI:** 10.3390/jcm15135192

**Published:** 2026-07-02

**Authors:** Nicolas L. Carayannopoulos, Puru Sadh, Zvipo M. Chisango, Siddharth Jasti, Michael J. Farias, Joseph E. Nassar, Jeffrey Okewunmi, Jinseong Kim, John Czerwein, Eren O. Kuris, Bryce A. Basques, Alan H. Daniels

**Affiliations:** Department of Orthopedic Surgery, Brown University, 1 Kettle Point Avenue, East Providence, RI 02914, USA; nlc90@scarletmail.rutgers.edu (N.L.C.); sadhpuru4@gmail.com (P.S.); zvipoc97@gmail.com (Z.M.C.); sidjasti4@gmail.com (S.J.); michael_farias@brown.edu (M.J.F.); jen06@mail.aub.edu (J.E.N.); jeffrey_okewunmi@brown.edu (J.O.); jkimmd726@gmail.com (J.K.); jczerwein@uoi.com (J.C.); eokuris@gmail.com (E.O.K.); brycebasques@gmail.com (B.A.B.)

**Keywords:** irritable bowel syndrome, spine surgery, postoperative outcomes, functional somatic syndromes, nociplastic pain, patient-reported outcomes

## Abstract

**Background/Objectives:** Irritable bowel syndrome (IBS) is among the most prevalent disorders of gut–brain interaction, yet its implications for spine surgery remain poorly characterized. This narrative review examines how IBS influences symptom presentation and postoperative outcomes in spine surgery patients. **Methods:** We synthesized the neurobiologic, epidemiologic, and perioperative literature linking IBS with musculoskeletal pain, spine-related symptomatology, and surgical outcomes, drawing on spine-specific data where available and on related surgical and chronic-pain populations where it was not. **Results:** IBS is characterized by central sensitization, impaired descending inhibition, increased temporal summation, autonomic dysregulation, and a high prevalence of psychiatric comorbidity, which manifest as widespread hyperalgesia and symptom amplification that overlap with pain mechanisms common in spine surgery patients. Epidemiologic studies indicate that patients with IBS undergo musculoskeletal and spinal procedures at disproportionately high rates, reflecting both symptom burden and diagnostic uncertainty from viscerosomatic overlap. These same factors have been associated with greater postoperative pain, elevated opioid requirements, slower functional recovery, and reduced satisfaction after spine surgery, although direct IBS-specific spine data remain limited. IBS may also confound preoperative assessment by mimicking radicular, discogenic, or sacroiliac pain. **Conclusions:** IBS represents an under-recognized potential modifier of symptom localization, perioperative pain trajectories, and functional recovery in spine surgery. Greater awareness of IBS-related nociplastic and psychosocial mechanisms may improve preoperative evaluation, risk stratification, perioperative management, and the design of future outcome studies.

## 1. Introduction

Irritable bowel syndrome (IBS) is a common disorder of the gut–brain interaction characterized by chronic abdominal pain and altered bowel habits, distinct from inflammatory bowel disease (IBD), which involves overt mucosal inflammation and structural pathology [1,2,3]. IBS is instead driven by altered sensory processing, central pain modulation, and dysregulated gut–brain signaling involving visceral hypersensitivity, autonomic dysfunction, and neuroimmune interactions [1,4].

Beyond gastrointestinal symptoms, IBS shows substantial overlap with chronic pain conditions, including headache disorders, temporomandibular disorders, and axial musculoskeletal pain [5,6]. Patients with IBS frequently demonstrate heightened pain sensitivity and symptom amplification, features that parallel pain-processing mechanisms increasingly recognized in chronic spinal pain populations [7]. Spinal pain is itself highly prevalent across general populations, shaped by a range of physical and psychosocial risk factors [8]. These shared characteristics underscore the potential relevance of IBS to perioperative outcomes in spine surgery [9].

Emerging evidence indicates that individuals with IBS may follow different clinical trajectories during musculoskeletal and spine evaluation when compared with patients without IBS [10]. Across orthopedic populations, functional somatic syndromes (FSSs), including IBS, are associated with higher postoperative pain, increased opioid use, and less improvement in patient-reported outcomes despite technically successful surgery [9,11]. FSS conditions are also strongly associated with psychiatric comorbidity and centralized pain phenotypes that independently predict worse postoperative pain and patient-reported outcomes across orthopedic procedures [5]. Despite these associations, IBS remains under-recognized as a potential modifier of surgical outcomes in spine care [6].

The purpose of this review is to synthesize current evidence on the epidemiology, mechanisms, and perioperative implications of IBS in spine surgery, with emphasis on pain trajectories, patient-reported outcomes, and practical considerations for surgical decision-making. Because IBS-specific spine surgery data are limited, we explicitly distinguish between findings derived from spine populations and those from related chronic-pain and surgical cohorts.

## 2. Materials and Methods

### 2.1. Search Strategy

This narrative review synthesizes the neurobiologic, epidemiologic, and perioperative literature linking IBS to spine surgery. We searched PubMed/MEDLINE (National Library of Medicine, Bethesda, MD, USA), Embase (Elsevier, Amsterdam, The Netherlands), Scopus (Elsevier, Amsterdam, The Netherlands), and the Cochrane Library (John Wiley & Sons, Hoboken, NJ, USA) from database inception through June 2026. Search terms combined controlled vocabulary and free-text keywords for the exposure (“irritable bowel syndrome,” “disorders of gut–brain interaction,” “functional somatic syndrome,” “visceral hypersensitivity,” “central sensitization,” “nociplastic pain”) with terms for the clinical context (“spine surgery,” “spinal fusion,” “laminectomy,” “discectomy,” “low back pain,” “postoperative pain,” “opioid,” “patient-reported outcomes,” “fusion,” “decompression”). Reference lists of relevant primary studies and prior reviews were hand-searched to identify additional sources.

### 2.2. Study Selection and Synthesis

We included English-language, peer-reviewed studies, systematic reviews, and mechanistic or epidemiologic reports relevant to IBS pathophysiology, the overlap between IBS and musculoskeletal or spinal pain, and perioperative or postoperative outcomes. Spine-specific studies were prioritized; where spine-specific data were unavailable, indirect evidence from other surgical and chronic-pain populations was included and is identified as extrapolative throughout the text.

## 3. Epidemiology

### 3.1. Basic Epidemiology

IBS is one of the most common disorders of the gut–brain system, with global prevalence estimates ranging from 10–11% in meta-analyses and approximately 5–10% in more recent population-based studies using contemporary Rome criteria [12,13]. Prevalence varies by region and diagnostic criteria but consistently shows a female predominance (OR~1.7) and higher rates among younger and middle-aged adults, with declining prevalence after age 50 [12,14]. Given the high prevalence of chronic low back pain in the general population, these demographic patterns suggest that a subset of patients presenting for spine evaluation may have comorbid IBS, whether formally diagnosed or not [6]. Subtype patterns also demonstrate sex differences, with the constipation-predominant subtype (IBS-C) more common among women and diarrhea-predominant (IBS-D) more common among men [15,16]. These differences may have perioperative relevance, particularly with respect to baseline bowel function, medication tolerance, and postoperative gastrointestinal risk [15,17].

Psychiatric comorbidity is a common feature of IBS populations. Up to one-third of IBS patients meet criteria for anxiety or depression, and meta-analytic data suggest a threefold higher risk for mood and anxiety disorders compared with healthy controls [18,19]. Longitudinal cohort studies further show bidirectional associations, with IBS increasing the risk of subsequent psychological distress and preexisting distress increasing the likelihood of IBS onset [20,21,22,23]. Importantly, similar psychosocial profiles, including anxiety, depression, and pain catastrophizing, are overrepresented among patients presenting for chronic spinal pain and are independently associated with worse postoperative pain and functional outcomes after spine surgery [24].

### 3.2. IBS and Increased Rates of Musculoskeletal Surgery

Several population-based studies indicate that patients with IBS undergo musculoskeletal and spine procedures at higher-than-expected rates. In a large analysis of 89,008 examinees, individuals with clinically diagnosed IBS showed significantly increased odds of undergoing back surgery (4.4% vs. 2.9% in controls), even after multivariable adjustment [10]. This study also reported higher rates of cholecystectomy and hysterectomy among IBS patients, reinforcing a broader pattern of elevated procedure utilization.

The high rate of surgical utilization may reflect diagnostic ambiguity, with visceral or centrally mediated pain sometimes misattributed to structural spine pathology. IBS-related hypervigilance, symptom amplification, and overlapping atypical pain-processing mechanisms can complicate attribution of discomfort and may contribute to higher rates of both spine consultations and operative interventions [7,25].

## 4. Pathophysiology Relevant to Spine Surgery

IBS is characterized by complex disturbances in visceral sensation, central pain modulation, autonomic function, and immune signaling [26]. Rather than representing isolated gastrointestinal pathology, these disturbances reflect a broader nociplastic pain phenotype with relevance to surgical pain outcomes [27,28]. From a perioperative perspective, these features may influence postoperative pain trajectories, sensitivity to surgical stimuli, and vulnerability to opioid-associated adverse effects following spine surgery [7,29]. In the context of spine surgery, these mechanisms contribute to three outcome domains: heightened central pain processing may amplify incisional and axial postoperative pain and opioid requirements; autonomic dysregulation may predispose to postoperative ileus and delayed gastrointestinal recovery; and viscerosomatic referral may complicate the localization of residual postoperative pain.

### 4.1. IBS and Chronic Low Back Pain Overlap

IBS is strongly associated with chronic low back pain (CLBP), with epidemiologic studies showing substantially higher rates of axial musculoskeletal symptoms among IBS patients compared with healthy controls [7]. Several mechanistic factors may account for this overlap, including viscerosomatic convergence, central sensitization, and shared psychosocial risk profiles [17,28]. Importantly, this association has implications for spine surgery evaluation: IBS-related musculoskeletal symptoms can mimic radiculopathy, sacroiliac joint pain, or nonspecific axial pain, increasing the risk of misclassification and potentially contributing to higher surgical utilization [10].

### 4.2. Visceral Hypersensitivity, Central Sensitization, and Nociplastic Pain

Visceral hypersensitivity is a hallmark abnormality in IBS, characterized by lowered sensory thresholds, enhanced activation of pain-processing regions, and abnormal engagement of spinal and supraspinal pathways [30]. Functional neuroimaging demonstrates exaggerated responses in the anterior cingulate cortex, insula, thalamus, and prefrontal regions during visceral stimulation [19,22], and experimental data show increased somatic pain sensitivity and greater after-sensations following noxious stimulation in IBS cohorts [25]. These observations reflect an atypical, centrally augmented pain-processing phenotype with diminished inhibitory control. Mechanistically, central sensitization in IBS involves increased excitability of dorsal horn neurons, expanded receptive fields, impaired descending inhibition, and enhanced responsiveness to afferent input [31,32], contributing to both visceral and somatic hyperalgesia [33]. IBS patients demonstrate impaired conditioned pain modulation and heightened temporal summation, reflecting compromised descending inhibitory pathways and exaggerated facilitation [34]. Across surgical disciplines, similar phenotypes have been associated with higher postoperative pain, delayed recovery, and reduced improvement in patient-reported outcomes, providing a plausible mechanistic framework through which IBS may influence spine surgery outcomes [35,36,37,38,39].

### 4.3. Autonomic Dysregulation and Viscerosomatic Reflexes

Autonomic dysfunction is frequently identified in IBS, characterized by reduced parasympathetic tone, heightened sympathetic responsiveness, and abnormal heart rate variability [24]. Viscerosomatic reflexes arising from chronic gastrointestinal irritation can produce referred pain, increased paraspinal muscle tone, and sensitization of spinal segments corresponding to the lower thoracolumbar and lumbosacral regions [40], which may explain why many IBS patients experience axial or pelvic pain that is difficult to localize and may be misinterpreted as originating from spinal structures [41]. These autonomic abnormalities also have perioperative relevance: heightened sympathetic tone may predispose IBS patients to postoperative ileus, nausea, and delayed return of bowel function, factors of particular concern in opioid-intensive spine surgery pathways [42].

### 4.4. Neuroimmune Interactions and the Gut–Brain Axis

Low-grade inflammation, altered microbiota composition, and impaired intestinal barrier function are frequently described in IBS [43,44,45]. These factors may influence nociceptive signaling via cytokine release, mast cell activation, or microbial metabolite-mediated neural sensitization [11,46,47], with increased mucosal immune activation near afferent nerve terminals correlating with visceral pain severity [47]. While these pathways are less directly established in spine surgery, they may contribute to variability in postoperative inflammation, analgesic responsiveness, and recovery trajectories. In related chronic-pain populations, lifestyle- and microbiome-directed interventions have been associated with changes in pain, suggesting a potentially modifiable gut–brain axis [48].

### 4.5. Overlap with Functional Somatic Syndromes

**Figure 1:**
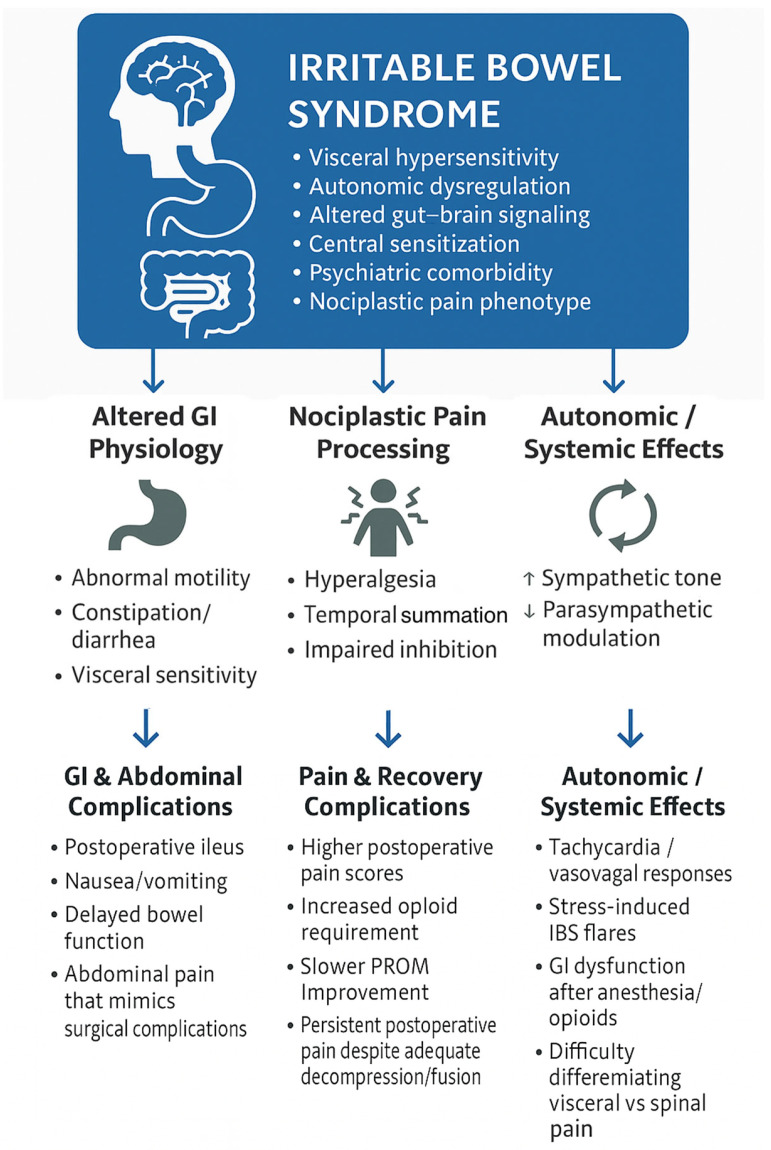
Summary of the pathophysiologic effects of irritable bowel syndrome relevant to spine surgery (original figure created by the authors). This represents a conceptual model: the upper-tier mechanisms (central sensitization, visceral hypersensitivity, autonomic dysregulation, and neuroimmune signaling) are established features of IBS supported by the studies cited below, whereas the lower-tier perioperative complications are associations extrapolated from non-spine populations. The mechanisms depicted are supported by the studies cited in Section 4: central sensitization and impaired descending inhibition [32,33,34]; visceral hypersensitivity and nociplastic pain [25,30,49]; autonomic dysregulation [24,42]; and neuroimmune and microbiome dysregulation [11,43,44,45,46,47]. Abbreviations: GI, gastrointestinal; IBS, irritable bowel syndrome; PROM, patient-reported outcome measure.

IBS frequently co-occurs with functional somatic syndromes such as fibromyalgia, chronic fatigue syndrome, migraine, and temporomandibular disorders [6]. These conditions share mechanistic features—central sensitization, dysautonomia, and impaired descending pain inhibition—and commonly predict worse postoperative outcomes across surgical fields [38]. Spine-specific data indicate that patients with FSS experience higher postoperative pain, slower improvement in patient-reported outcome measures (PROMs), and greater opioid requirements following lumbar or cervical procedures [5]. Given this mechanistic convergence, it is plausible—though not yet directly demonstrated—that IBS may similarly modulate spine surgery recovery (Figure 1).

## 5. Preoperative Considerations

Patients with IBS often present with complex symptom profiles, including overlapping visceral and somatic pain, psychiatric comorbidity, and heightened pain sensitivity [6], which may complicate attribution of symptoms to structural spinal pathology. IBS-related abdominal or pelvic discomfort can present with back or sacroiliac pain patterns that resemble discogenic or radicular symptoms, increasing the risk of diagnostic uncertainty.

Psychological comorbidity—including anxiety, depression, catastrophizing, and somatic hypervigilance—is significantly more common in IBS and independently predicts worse postoperative pain and slower recovery across surgical specialties [19]. These factors may influence both expectations and perceived outcomes in spine surgery. IBS also frequently coexists with altered central pain modulation and functional somatic syndromes, amplifying somatic pain responses and reducing responsiveness to standard analgesic pathways [5]; affected patients may benefit from preoperative counseling that emphasizes central pain mechanisms, expectation management, and targeted multimodal strategies.

Medication history warrants careful review. Many IBS patients use antispasmodics, tricyclic antidepressants (TCAs), selective serotonin or serotonin–norepinephrine reuptake inhibitors (SSRIs/SNRIs), anticholinergic agents, and dietary supplements, each of which may interact with perioperative analgesics, anesthesia, or bowel function [41,50]. Chronic laxative use, opioid sensitivity, and baseline constipation risk should be identified proactively.

See Table 1 for a summary of preoperative medication recommendations [41,50].

## 6. Postoperative Outcomes and Pain Trajectories

Evidence from the musculoskeletal and spine literature suggests that patients with FSS, including those with IBS, experience higher postoperative pain levels, increased opioid use, and slower functional improvement [5]. In spine cohorts, FSS has been associated with diminished PROM gains following both fusion and decompression procedures, suggesting that central pain-processing characteristics influence postoperative recovery [5].

Patients with IBS often report heightened somatic pain, greater sensitivity to mechanical stimuli, and larger after-sensations following surgical incisions or postoperative rehabilitation [33], which may contribute to prolonged pain trajectories and lower satisfaction even when radiographic or structural outcomes are favorable. Psychological factors such as catastrophizing and fear-avoidance may further contribute to functional limitations. Although direct IBS-specific data in spine surgery are limited, the consistency of findings across the chronic-pain and orthopedic literature supports the plausibility of similar patterns in IBS patients undergoing spine procedures.

See Table 2 for a summary of complications potentially associated with IBS in spine surgery patients, annotated by the predominant type of supporting evidence [5,11,43,44,45,46,47].

### Perioperative Pain Management

Opioid-sparing multimodal analgesia is important in IBS patients given the risk of narcotic bowel syndrome and baseline gastrointestinal dysfunction. Consensus guidelines emphasize multimodal analgesia for patients with chronic pain conditions, and combinations of acetaminophen, nonsteroidal anti-inflammatory drugs, and adjuncts such as gabapentinoids reduce 24-h opioid consumption and pain scores after spine surgery. In a network meta-analysis of randomized trials in adult spine surgery, multimodal triple-drug therapy (acetaminophen, a nonsteroidal anti-inflammatory drug, and an adjunct such as a gabapentinoid) was the most effective regimen, with pooled reductions of approximately 26 mg in 24-h morphine-equivalent consumption and 2.3 points in pain score [51]. Specific strategies include ketamine infusions for opioid-tolerant patients, regional techniques such as intrathecal morphine and fascial-plane blocks, and preemptive analgesia, alongside proactive bowel-management protocols and continuation of baseline IBS medications (TCAs, SSRIs/SNRIs, antispasmodics) throughout the perioperative period. These recommendations derive from general enhanced-recovery and multimodal-analgesia evidence rather than from IBS-specific perioperative trials.

## 7. Patient-Reported Outcomes and Satisfaction

PROMs in IBS patients undergoing spine surgery may be influenced by baseline pain severity, generalized hyperalgesia, comorbid mood disorders, and expectations about surgical relief [19]. Studies of FSS demonstrate that such patients often experience less improvement in PROMs despite similar technical success [5]. In shoulder and elbow surgery, patients with FSS (including IBS) showed significantly lower postoperative Disabilities of the Arm, Shoulder and Hand, American Shoulder and Elbow Surgeons, and quality-of-recovery scores compared with controls, though PROMs still improved from baseline in most patients [11]. Direct spine-specific evidence is consistent with these findings. In a prospective cohort undergoing lumbar decompression, preoperative central sensitization (Central Sensitization Inventory ≥ 40) independently predicted smaller three-month improvements in disability and pain [52].

In spine surgery specifically, achievement of the minimal clinically important difference (MCID) is reduced in patients with psychological distress and centralized pain phenotypes. Patients with depression have worse baseline Oswestry Disability Index (ODI) scores (51.9 vs. 36.4 in a lumbar decompression cohort, *p*-value < 0.001). Although they achieve a similar magnitude of ODI improvement, they remain significantly more disabled postoperatively (37.0 vs. 23.4, *p*-value < 0.001) [53]. Reported risk factors for failing to reach the MCID after elective lumbar spine surgery include chronic opioid use (relative risk 1.23–1.25), symptom duration longer than one year (RR 1.34–1.41), and psychological distress [54]. PROM-based assessments should therefore be interpreted in the context of IBS-related symptom patterns, with attention to psychological and nociplastic contributors; while patients with FSS may have lower absolute PROM scores postoperatively, many still experience clinically meaningful improvement [11].

## 8. Insights from Other Orthopedic Specialties

Evidence from total joint arthroplasty, shoulder, and elbow surgery indicates that patients with IBS or other FSS demonstrate worse postoperative pain control, greater opioid consumption, longer recovery times, and lower physical-function scores compared with controls, underscoring the broader relevance of pain-amplification phenotypes and gut–brain dysregulation across musculoskeletal surgery [11]. Although these findings cannot be directly extrapolated to spine surgery, they support the concept that IBS-related neurophysiologic alterations may negatively affect postoperative musculoskeletal outcomes more broadly.

## 9. Practical Clinical Implications for Spine Surgeons

Clinicians evaluating IBS patients for spine surgery should conduct careful symptom localization, ensuring that visceral or centrally mediated pain is not misinterpreted as spinal pathology; this may reduce the risk of low-value interventions and help ensure appropriate patient selection. Preoperative screening for psychiatric comorbidity, maladaptive pain processing, and symptom amplification may help identify patients at higher risk for poor pain outcomes, and multimodal prehabilitation, expectation management, and coordination with gastroenterology or behavioral health may optimize care. Postoperatively, emphasis on opioid-sparing analgesia, early mobilization, bowel-management protocols, and close monitoring of stress-related symptom exacerbation is recommended.

### Shared Decision-Making and Expectation Management

Patients with IBS may benefit from preoperative counseling that emphasizes the role of central pain mechanisms in their symptom profile, realistic expectations regarding postoperative pain trajectories, and the importance of multimodal rehabilitation. Coordination with gastroenterology or behavioral health services may optimize outcomes in complex cases. While IBS patients may experience slower PROM improvement and higher pain scores, many still achieve clinically meaningful benefit from appropriately indicated spine surgery.

## 10. Discussion

This review synthesizes mechanistic, epidemiologic, and perioperative evidence indicating that IBS is associated with a centralized, nociplastic pain phenotype that may influence spine surgery outcomes. Three observations are reasonably well supported: IBS involves central sensitization, impaired descending inhibition, autonomic dysregulation, and frequent psychiatric comorbidity. Patients with IBS undergo musculoskeletal and spinal procedures at relatively high rates, and functional somatic syndromes, of which IBS is one, are associated with worse pain, higher opioid use, and lower PROM scores across orthopedic surgery. The contention that IBS specifically worsens spine surgery outcomes, by contrast, remains a mechanistically grounded hypothesis rather than a directly demonstrated finding.

### 10.1. Relationship to Prior Work and Novel Contribution

**Figure 2:**
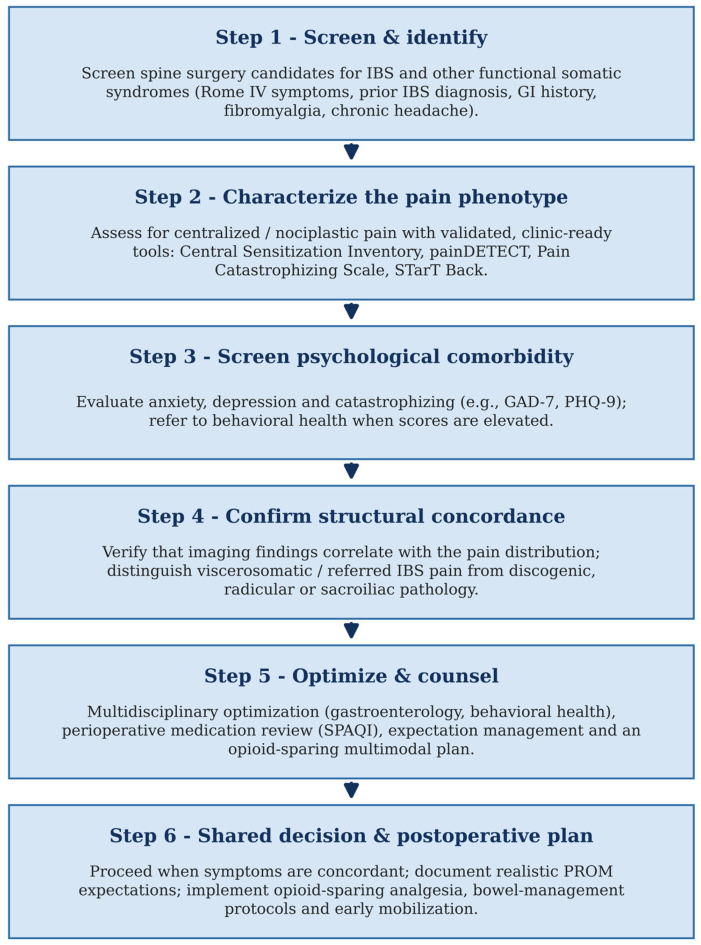
Proposed framework for the preoperative assessment of spine surgery candidates with comorbid or suspected IBS. The framework is a conceptual synthesis of the considerations discussed in Section 5 and Section 9 and is intended to structure evaluation and counseling rather than to serve as a validated decision rule. Abbreviations: GAD-7, Generalized Anxiety Disorder-7; GI, gastrointestinal; IBS, irritable bowel syndrome; PHQ-9, Patient Health Questionnaire-9; PROM, patient-reported outcome measure; SPAQI, Society for Perioperative Assessment and Quality Improvement.

Previous reviews have addressed functional somatic syndromes broadly in orthopedic surgery, or the gastrointestinal management of IBS in isolation. To our knowledge, this is the first review to integrate the nociplastic-pain physiology of IBS with spine-surgery-specific preoperative evaluation, perioperative medication management, and patient-reported outcomes within a single framework. Its principal contribution is translational: it reframes IBS from a purely gastrointestinal comorbidity into a potential modifier of pain phenotype and surgical expectation, and it offers an actionable, stepwise approach to preoperative assessment (Figure 2). This framing may improve patient selection, sharpen informed consent, and define concrete hypotheses for future prospective study.

### 10.2. Distinguishing Confounding from Mechanism

Several factors that travel with IBS, depression, anxiety, pain catastrophizing, sleep disturbance, and other functional somatic syndromes such as fibromyalgia and chronic headache, are established predictors of worse spine surgery outcomes. Sleep disruption in particular is closely linked to central sensitization and pain amplification in related musculoskeletal populations [55]. This contributes to difficulty in attributing any outcome effect to IBS rather than to its psychological and somatic comorbidities, which may lie on a shared pathway of central sensitization or act as independent confounders. Until studies adjust for these covariates, associations between IBS and spine outcomes should be regarded as hypothesis-generating.

### 10.3. Clinically Applicable Assessment Tools

Translating these mechanisms into practice requires instruments that spine surgeons can use in the clinic. Centralized and nociplastic pain can be screened with the Central Sensitization Inventory and painDETECT; pain-related psychological risk can be captured with the Pain Catastrophizing Scale and the STarT Back Screening Tool; and mood comorbidity can be screened with the GAD-7 and PHQ-9. Incorporating one or more of these validated, brief tools into the preoperative pathway would allow IBS-related central pain features to be identified and addressed alongside structural assessment.

### 10.4. Limitations

Although the search was broad and structured, it did not apply formal risk-of-bias assessment or quantitative synthesis, and selection bias cannot be excluded. Much of the evidence linking IBS to spine outcomes is indirect, drawn from other surgical or chronic-pain populations and from mechanistic studies. Moreover, much of this indirect evidence derives from the broader functional somatic syndrome literature. Because IBS is not interchangeable with fibromyalgia, chronic fatigue syndrome, or other overlapping conditions, extrapolation from this literature to IBS patients undergoing spine surgery should be made with caution. Heterogeneity in IBS diagnostic criteria across decades of the literature, the absence of prospective spine cohorts with validated IBS ascertainment, and the confounding discussed above limit causal inference. The proposed framework in Figure 1 and Figure 2 is conceptual and has not been prospectively validated.

### 10.5. Future Directions

Priorities for future research include prospective spine surgery cohorts that incorporate validated central-sensitization and psychological measures. Evaluation of opioid-sparing, multidisciplinary perioperative pathways in IBS patients and prospective testing of the preoperative framework proposed here against pain, opioid, PROM, and satisfaction endpoints is warranted.

## 11. Conclusions

IBS is a common and complex disorder of gut–brain interaction with potential relevance to spine surgery. Its associated abnormalities in pain processing, autonomic function, and psychological comorbidity may influence perioperative gastrointestinal complications, pain trajectories, and postoperative satisfaction. Although IBS-specific spine data remain limited and current associations are largely indirect, converging evidence from pain neuroscience, musculoskeletal surgery, and functional somatic syndromes suggests that IBS may act as a modifier of surgical outcomes. Improved recognition of IBS-related factors may assist with risk stratification, shared decision-making, and tailored perioperative management, and prospective studies are needed to test these associations directly and to define evidence-based strategies for this population.

## Figures and Tables

**Table 1:** Preoperative medication recommendations based on Society for Perioperative Assessment and Quality Improvement (SPAQI) consensus statements [41,50].

Medication Class	Examples	Perioperative Recommendation	Timing Guidance
Secretagogues (chloride channel activators)	Lubiprostone	Continue	Take up to and including day of surgery
Guanylate cyclase-C agonists	Linaclotide, plecanatide	Continue	Take up to and including day of surgery; resume when enteral intake tolerated
Serotonergic neuroenteric modulators	Tegaserod, prucalopride, alosetron	Continue	Take up to and including day of surgery; monitor QT/ischemia risks where applicable
Laxatives (osmotic, stimulant, softener)	PEG 3350, lactulose, senna, bisacodyl, Mg salts, docusate	Hold morning of surgery	Last dose evening prior; skip morning dose unless bowel prep required
Antispasmodics/anticholinergics	Dicyclomine, hyoscyamine	Hold morning of surgery	Take last dose night before; omit morning dose due to anticholinergic effects
μ-Opioid modulators (IBS-D)	Eluxadoline	No clear SPAQI guideline; typically hold morning of surgery (institution dependent)	Coordinate with anesthesia; resume once PO and low risk for pancreatitis/SOD spasm
TCAs for pain modulation	Amitriptyline, nortriptyline	Continue	Continue through surgery; taper only if high arrhythmia/QT risk
SSRIs	Sertraline, fluoxetine, citalopram, paroxetine	Continue	Discontinuation syndrome risk when stopped abruptly
SNRIs	Duloxetine, venlafaxine	Continue	Discontinuation syndrome risk when stopped abruptly
Anti-diarrheal agents	Loperamide, diphenoxylate/atropine	Hold morning of surgery	Hold morning dose if obstruction/ileus suspected
Non-prescription IBS adjuncts	Psyllium fiber, peppermint oil	Continue	Hold fiber on morning of surgery if aspiration risk or severe GI dysmotility
Antibiotics used in IBS-D/SIBO overlap	Rifaximin	Continue	Take as scheduled; no perioperative restrictions

Abbreviations: GI, gastrointestinal; IBS-D, diarrhea-predominant irritable bowel syndrome; Mg, magnesium; PEG, polyethylene glycol; PO, per os (by mouth); QT, QT interval; SIBO, small intestinal bacterial overgrowth; SNRIs, serotonin–norepinephrine reuptake inhibitors; SOD, sphincter of Oddi; SPAQI, Society for Perioperative Assessment and Quality Improvement; SSRIs, selective serotonin reuptake inhibitors; TCAs, tricyclic antidepressants.

**Table 2:** Complications potentially associated with IBS in patients undergoing spine surgery.

Complication Category	Specific Complication	Rationale/Evidence Linking IBS to Higher Risk	Predominant Evidence Basis
Gastrointestinal & abdominal	Postoperative ileus (POI)	IBS listed as a candidate POI risk factor in spine cohorts; autonomic dysregulation, heightened sympathetic tone, and baseline motility disturbance.	Indirect (spine cohort association)
	Postoperative nausea/vomiting (PONV)	Increased visceral hypersensitivity and vagal dysfunction; higher perioperative nausea in GI literature extrapolated to spine pathways.	Indirect (extrapolated)
	Delayed return of bowel function	Baseline constipation, chronic laxative use, autonomic imbalance, and opioid sensitivity increase risk of delayed GI recovery.	Mechanistic/extrapolated
	Abdominal pain mimicking complication	IBS flares may present as postoperative abdominal pain, complicating differentiation from true abdominal pathology.	Mechanistic
Opioid-related adverse events	Increased opioid requirement	IBS and other FSS show higher postoperative opioid consumption across musculoskeletal procedures.	Indirect (FSS musculoskeletal data)
	Opioid intolerance/hypersensitivity	Central sensitization results in heightened pain reporting and lower opioid efficacy, leading to prolonged or escalated use.	Mechanistic
	Opioid-induced constipation exacerbation	IBS-C and mixed-type IBS patients are at elevated risk for postoperative constipation.	Mechanistic
Pain & recovery	Higher postoperative pain scores	Generalized hyperalgesia, impaired descending inhibition, and enhanced temporal summation predict worse surgical pain trajectories.	Indirect (chronic-pain data)
	Slower or blunted PROM improvement	FSS (including IBS) consistently predict lower PROM gains following spine surgery.	Indirect (FSS spine data)
	Persistent postoperative pain	Nociplastic pain mechanisms worsen recovery independent of structural outcomes.	Mechanistic
Surgical & mechanical (indirect)	Higher perceived failure despite adequate decompression/fusion	Pain amplification can lead to dissatisfaction even after technically successful procedures.	Mechanistic
	Increased return visits or imaging	Diagnostic ambiguity from visceral–somatic overlap may prompt more postoperative evaluation.	Mechanistic
Systemic/physiologic stress	Autonomic instability (tachycardia, vasovagal)	Heightened sympathetic reactivity and reduced parasympathetic tone may affect intra- and postoperative hemodynamics.	Mechanistic
	Postoperative exacerbation of IBS symptoms	Surgical stress, anesthesia, perioperative opioids, and bowel manipulation can provoke flares.	Mechanistic
Rare reported events	Abdominal complications post-posterior fusion	Case reports describe IBS as a comorbidity in bowel obstruction or severe ileus after posterior procedures (no causal link established).	Case-level
	Misclassification of IBS pain as recurrent spinal pathology	IBS-induced pelvic/low-back pain may resemble radicular or discogenic pain, leading to unnecessary intervention.	Conceptual

Abbreviations: FSS, functional somatic syndrome; GI, gastrointestinal; IBS, irritable bowel syndrome; IBS-C, constipation-predominant IBS; POI, postoperative ileus; PONV, postoperative nausea and vomiting; PROM, patient-reported outcome measure. Entries are derived from (1) spine surgery cohorts listing IBS as a comorbidity or candidate risk factor, (2) studies of postoperative ileus after spine surgery, (3) case series describing abdominal complications after spine fusion, and (4) systematic reviews of functional somatic syndromes including IBS [5,11,43,44,45,46,47].

## Data Availability

No new data were created or analyzed in this study. Data sharing is not applicable to this article.

## Abbreviations

The following abbreviations are used in this manuscript:

CLBP	chronic low back pain
CSI	Central Sensitization Inventory
FSS	functional somatic syndrome
GI	gastrointestinal
IBD	inflammatory bowel disease
IBS	irritable bowel syndrome
IBS-C/IBS-D	constipation-/diarrhea-predominant IBS
MCID	minimal clinically important difference
ODI	Oswestry Disability Index
POI	postoperative ileus
PONV	postoperative nausea and vomiting
PROM	patient-reported outcome measure
SPAQI	Society for Perioperative Assessment and Quality Improvement
SNRIs/SSRIs	serotonin–norepinephrine/selective serotonin reuptake inhibitors
TCAs	tricyclic antidepressants
